# Nonlinearities Lead to Qualitative Differences in Population Dynamics of Predator-Prey Systems

**DOI:** 10.1371/journal.pone.0062530

**Published:** 2013-04-25

**Authors:** Olga M. C. C. Ameixa, Gerben J. Messelink, Pavel Kindlmann

**Affiliations:** 1 Department of Biodiversity Research, Global Change Research Centre, České Budějovice, Czech Republic; 2 Wageningen UR Greenhouse Horticulture, Bleiswijk, The Netherlands; 3 Institute for Environmental Studies, Charles University, Prague, Czech Republic; University of California, Berkeley, United States of America

## Abstract

Since typically there are many predators feeding on most herbivores in natural communities, understanding multiple predator effects is critical for both community and applied ecology. Experiments of multiple predator effects on prey populations are extremely demanding, as the number of treatments and the amount of labour associated with these experiments increases exponentially with the number of species in question. Therefore, researchers tend to vary only presence/absence of the species and use only one (supposedly realistic) combination of their numbers in experiments. However, nonlinearities in density dependence, functional responses, interactions between natural enemies etc. are typical for such systems, and nonlinear models of population dynamics generally predict qualitatively different results, if initial absolute densities of the species studied differ, even if their relative densities are maintained. Therefore, testing combinations of natural enemies without varying their densities may not be sufficient. Here we test this prediction experimentally. We show that the population dynamics of a system consisting of 2 natural enemies (aphid predator *Adalia bipunctata* (L.), and aphid parasitoid, *Aphidius colemani* Viereck) and their shared prey (peach aphid, *Myzus persicae* Sulzer) are strongly affected by the absolute initial densities of the species in question. Even if their relative densities are kept constant, the natural enemy species or combination thereof that most effectively suppresses the prey may depend on the absolute initial densities used in the experiment. Future empirical studies of multiple predator – one prey interactions should therefore use a two-dimensional array of initial densities of the studied species. Varying only combinations of natural enemies without varying their densities is not sufficient and can lead to misleading results.

## Introduction

Most studies of predator-prey interactions have considered relationships between a single prey species and a single predator species [1]. However, in natural communities there are typically many predators feeding on most species of prey [2], [3]. Understanding multiple predator effects is therefore critical for both community [4], [5] and applied ecology – e.g., in biological control programs, predicting outcomes of multiple predator – single prey interactions is especially important [6], [7], [8], since interactions between introduced predators or parasitoids and other natural enemies may even inadvertently increase their prey (pest) populations [9], 10,11,12,13. However, discussion so far has not yielded any clear-cut results, mainly because the observed population dynamics in multiple predator – single prey communities are often quite complicated due to several types of nonlinear effects, which, generally predict qualitatively different results, if initial absolute densities of the species studied differ, even if their relative densities are maintained [14], [15], [16], [17].

Most commonly mentioned in this context are nonlinear predator effects (interactions among predators), which can raise (risk enhancement) or lower (risk reduction) a prey’s risk of predation in the presence of multiple predator species [18], [19]. Whether nonlinear outcomes are present in the form of risk enhancement or risk reduction will influence whether the prey’s population growth rates are higher or lower than those predicted by linear predator effects (e.g., [20], [21]). The reasons behind nonlinear predator effects occurrence include intraguild predation, competition or changes in behaviour (e.g., oviposition, feeding) of the predators. There is little doubt that such interactions occur in natural communities [22], [23], [24], [25], [26], [27]. However, there is still uncertainty about their effect on population densities of the species in question [25], [28].

One nice example of risk reduction is Rosenheim et al. [29] who document the effects of multiple predators on aphid population growth rates over several generations. In the absence of predators, aphid populations increased dramatically. In the presence of predatory lacewings (*Chrysoperla*) aphid population growth was suppressed. However, when the predatory bugs (*Geocoris*, *Nabis* and *Zelus*) were also present, aphids did well, even though these bugs can eat aphids. The reason for this is that the bugs killed the lacewings and thus released the aphids from the regulatory effect of lacewing predation.

Also other nonlinear effects such as nonlinear functional and numerical responses, density dependence (intraspecific competition) within the species in question, prey density, among others, may contribute to the complexity of the outcomes. For example, prey density is widely understood to influence mortality rates caused by single predator species [30], [31], [32], [33] and there is evidence suggesting that the predation risk from multiple predator species may also be influenced by prey density. For example, Losey and Denno [20] reported that the strength of risk enhancement for aphids in the presence of one foliar-foraging and one ground-foraging predator increased with prey density. The nonlinearity occurs, if the intensity of interspecific interactions between predators changes with prey density, which is known to happen [34]. For example, intraguild predation and other interspecific interactions have been found between ladybird beetles of different species at low aphid (prey) density but not at high prey density [35], [36]. Also the results obtained by Griffen [37] indicate that the strength of multiple predator effects (both risk reduction and risk enhancement) can vary with prey density.

Experimental studies of multiple predator effects on prey populations like that of Rosenheim et al. [29], although very useful, are extremely demanding. The number of treatments and therefore also the time required for these experiments increases exponentially with the number of species: with one predator and one prey species 2 treatments are needed (one with predator and prey and one with prey alone), while with *n* predator species and one prey species 2*^n^* treatments are needed, just to include all possible combinations of presence/absence of each predator species, and in addition it is necessary to have several replicates of each treatment.

However, even these 2*^n^* treatments may not be sufficient, because the result of each treatment describes the population dynamics of a system consisting of the chosen combination of predators and prey only for one combination of their densities. If the aim is, for instance, only to show that the effect of many different predators deviates from the expectations based on the assumption that many different predators have additive or multiplicative, linear effects, then this is enough. If the aim, however, is to decide whether one or more natural enemies should be used to suppress a pest species in a real situation, then it may be necessary for the effect of more than one initial density of all species to be checked, especially if the interactions in the system are nonlinear: if, for example, doubling the initial densities of all species does not result in a doubling of their densities later in time.

In this paper, we demonstrate that in studies of multiple natural enemy effects on suppression of their prey population it is necessary to check for the effect of more than one initial density of all species. We used a system consisting of one aphid, one predator and one parasitoid species. As in many other experiments, our treatments consisted of only predators, only parasitoids and a combination of both predators and parasitoids, maintaining the total number of natural enemies constant across all treatments. Contrary to most other experiments, however, we then repeated the experiment with initial numbers of all species 3, 6, and 10 times larger, thus keeping their relative densities constant, while varying their absolute densities. Our goal was to see if this change in the absolute densities while keeping the ratios constant would affect the outcome of the experiment – the “winner”, i.e., the natural enemy species or their combination, which will most efficiently suppress the prey population.

The crucial message of this study is that the outcome of such experiments in multiple predator – one prey systems is strongly affected by initial densities of the species involved and we attribute this to nonlinearities in the system. As nonlinearities are typical for predator-prey systems – e.g., in intraspecific interactions and functional and numerical responses [14], , the message of this paper seems to apply to a broad range of systems.

## Materials and Methods

### Organisms

Aphids are a good model system for our study, as they are attacked by a large guild of endoparasitoids and predators [38], [39] Predators such as Coccinellidae do not to seem to distinguish parasitized from unparasitized aphids [40] and therefore they are frequently seen consuming aphid mummies [41]. Interactions between predator and parasitoid species can be direct, when one species eats another – e.g., a predator feeding on parasitized aphids, or indirect, when by reducing aphid abundance, predators indirectly affect parasitoid reproductive opportunities – exploitation competition [42]. Also, indirect interactions can arise through chains of direct interactions or because the presence of one species modifies the nature of the interaction between two others [43].

The plant, aphid, parasitoid and predator species used in our experiments were as follows:

- sweet pepper plants (*Capsicum annuum* L. cv. Ferrari) were grown by a commercial plant propagator in peat 20±1°C and 16L: 8D without application of insecticides. Single plants were planted in 4.4 l pots (21 cm diameter) with peat and placed in cages for the greenhouse trial. A standard nutrient solution for pepper plants was supplied twice a week by hand. This solution (pH = 5.5) was composed by (mmol/m^3^): NH_4_ (1.250), K (7.632), Ca (7.285), SO_4_ (2.386), Mg (2.081), NO_3_ (21.136), P (1.705), Fe (15.000), B (30.000), Cu (0.750), Mo (0.500), Zn (5.000) and Mn (10.000).
- red phenotypes of *Myzus persicae* (Sulzer) from the stock cultures at Wageningen UR Greenhouse Horticulture that were reared at 20±2°C and 16L:8D on sweet pepper plants.
- the parasitoid, *Aphidius colemani* Viereck mummies (i.e., parasitoid-immobilized aphids containing a well-developed parasitoid) were kindly supplied by Koppert Biological Systems. After emergence, the parasitoids were placed in individual tubes with a droplet of honey, to be more easily identified regarding their sex. After identification, the females were introduced into experimental cages together with the same number of males (1∶1) in order to maximize their chance of mating.
- the predators, *Adalia bipunctata* (Linnaeus) pupae were obtained from Entocare, Wageningen, The Netherlands. After emergence and when their cuticles had hardened, females and males were transferred to plastic boxes containing a piece of corrugated filter paper. They were kept at 20±2°C, 16L:8D. Each day, the boxes were cleaned and fresh aphids supplied. Female ladybirds selected for the experiments were between 15 and 25 days old, at which time they are sexually mature. In order to standardize hunger, females were deprived of food for 12 hours overnight before the beginning of the experiments.

### Experimental Design

A glasshouse experiment with standardized climatic conditions 20±2°C, 16L:8D, was undertaken in 60*60*90 cm cages covered with 0.6 mm mesh and with a zipper opening on one side.

Thirty six sweet pepper plants, which were on average 40 cm high, were infested with first instar *Myzus persicae*, which did not immediately start to reproduce and caged individually (one plant per cage). Nine plants were each infested with 40 aphids, 9 with 120, 9 with 240 and 9 with 400. The aphids were allowed to establish on the plants for 15±1 days. After that, different combinations of mated *Adalia bipunctata* and/or *Aphidius colemani* females were released inside the cages as recorded in Table 1. We used a 4×3×3 substitutive experimental design, thus there were four treatments, differing in absolute, but not in relative densities of the aphids and their natural enemies: the ratios of numbers of aphids, parasitoids and predators were kept constant within each treatment, but their absolute numbers were different (Table 1). In each treatment we had 3 sub-treatments (“Predators” – aphids together with the predator, “Predators+Parasitoids” – aphids together with both the predator and the parasitoid and “Parasitoids” – aphids together with the parasitoid). There were 3 replicates of each of these 12 sub-treatments. Thus there were 36 cages containing predators and/or parasitoids (Table 1). The current experiment did not include a no-predator (aphids only) treatment because it would not make logical sense to do so: the controls for a multiple predator experiment are the individual predator treatments [18], [44], [45], [46].

**Table 1:** Initial numbers of aphids, parasitoids (*Aphidius colemani*) and predators (*Adalia bipunctata*) used in the experiment.

Sub-treatment	Initial aphiddensities	A. bipunctata	A. colemani
**Predator**	40	2	0
	120	6	0
	240	12	0
	400	20	0
**Predator+Parasitoid**	40	1	1
	120	3	3
	240	6	6
	400	10	10
**Parasitoid**	40	0	2
	120	0	6
	240	0	12
	400	0	20

There were 3 replicates for each treatment (initial aphid density) in each sub-treatment (“Predators”, “Predators+Parasitoids” and “Parasitoids”).

After 7 days and subsequently twice a week, aphids were counted in the cages on 8 leaves selected at random; 4 on the upper part of the plant and 4 on the lower part. This was continued for 3 weeks, giving a total of 6 counts.

### Statistical Analysis

Repeated measures two-way ANOVA models were used with treatment (initial density) and sub-treatment (predator/predator+parasitoid/parasitoid) as between-subject factors and time as within-subject factor, the response variable was the number of aphids and the per capita effect magnitude, the last one was calculated according to Schmitz [46]. We applied the Greenhouse-Geisser (G–G) adjustment for tests of within-subject effects when the sphericity assumption was not met. This analysis was followed by pairwise comparisons among treatments using the Bonferroni-adjusted level of significance.

To test which combination was more effective we also used one-way ANOVA, using the average maximum number of aphids as the response variable. The one-way ANOVAs were calculated for each of the initial aphid densities used (40, 120, 240, and 400) separately. The results were compared using Duncan tests.

The data were always log transformed (y = ln(x+1)) prior all the analyses to meet statistical assumptions.

## Results

**Figure 1:**
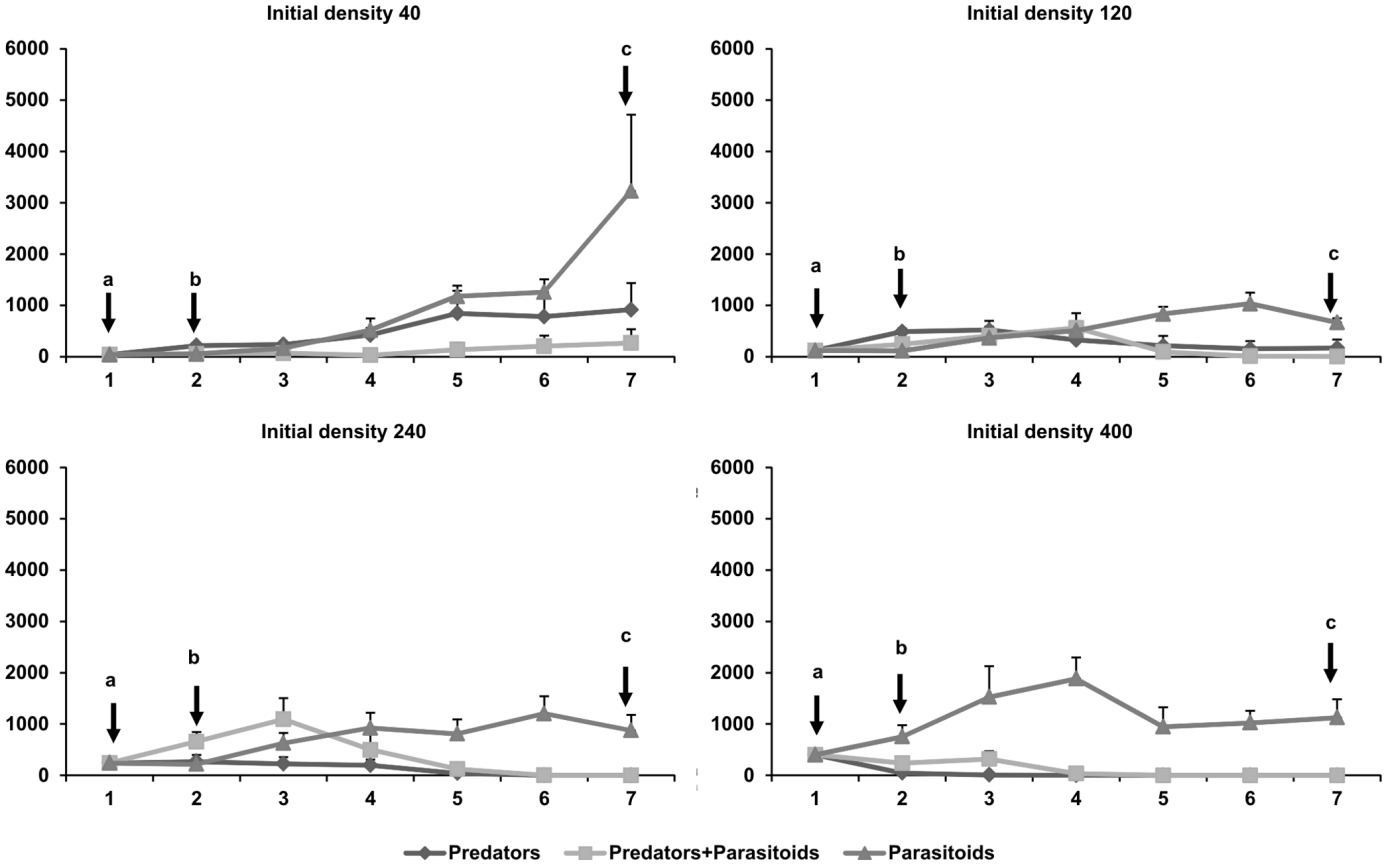
Average (±SE) numbers of aphids recorded in the different sub-treatments started with different initial numbers of aphids (40, 120, 240 and 400). Arrows indicate: a - first count, b - appearance of first larvae and mummies and c - final count.

**Figure 2:**
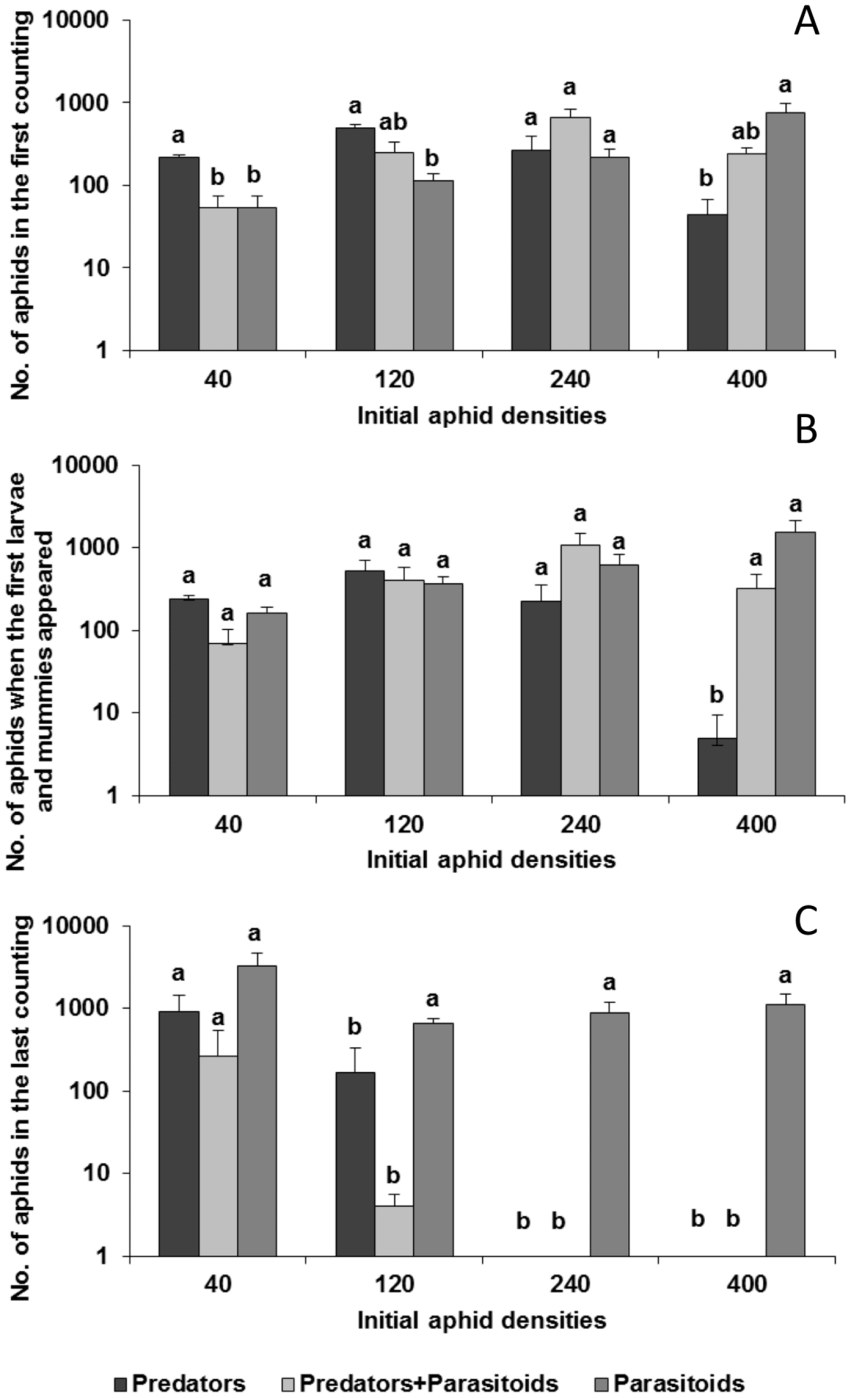
Average (±SE) number of aphids recorded at the first count (A), at the instant when the first mummies and larvae appeared (B) and in the last count (C). For each of the initial number of aphids used (40, 120, 240, 400 - indicated on the horizontal axis), different letters represent differences between the means recorded in the different sub-treatments.

Generally, the “Parasitoids” sub-treatment affected aphid population dynamics less than “Predators” sub-treatment or “Predators+Parasitoids” sub-treatment (Figs. 1 and 2B). At the lowest initial density (40) the aphid numbers continuously increased in the “Parasitoids” sub-treatment (Fig. 1). Aphid numbers started to decrease after the fifth count in density 120 in the “Parasitoids” sub-treatment (Fig. 1). For the highest initial densities of aphids (240, 400), there was an increase up until the third count in the “Parasitoids” sub-treatment (Fig. 1). In the “Predators” sub-treatment, except in the sub-treatment with initial density 40, the number of aphids was always very low, or the aphids were completely suppressed (Fig. 1, 2). Thus, the results indicate that the outcome strongly depends on the duration of the experiment.

In the sub-treatment in which there were the two natural enemies (“Predators+Parasitoids” sub-treatment) there were very low numbers of aphids in the treatment density 40, even if after the third count there was a slight increase in aphid numbers (Figs 1and 2). In the treatment density 120 there was a slight increase until the third count and for density 240 until the second count, but in these two treatments the densities then decreased and in density 240 by the fifth count there were no aphids present (Figs 1, 2). In the treatment density 400, there was a decrease after the second count and there were no aphids present at the fourth count (Fig. 1).

The two-factor repeated measures ANOVA of the number of aphids (Table 2) revealed that the type of sub-treatment had a significant effect on aphid abundance, the “Predators” and “Predators+Parasitoids” sub-treatments were more effective than the “Parasitoids” treatment. There were no differences between “Predators” and “Predators+Parasitoids” sub-treatments. The initial densities also had a marginal effect on aphid abundance (*P* = 0.051). The within-subjects effects analysis was significant for time, for the interaction of time with sub-treatment and for time with initial densities. Surprisingly, the interaction density by sub-treatment by time, was not significant.

**Table 2:** Repeated-measures two-way ANOVA of the recorded aphid numbers with treatment (initial aphid densities) and sub-treatment (“Predators”, “Predators+Parasitoids” and “Parasitoids”) counted as main effects.

Source	Type III Sum of Squares	df*	Mean Square	F	Sig.
*Tests of Between-Subjects Effects*					
Intercept	3860.029	1	3860.029	429.301	**<0.0001**
Treatment	80.484	3	26.828	2.984	**0.051**
Sub-treatment	517.258	2	258.629	28.764	**<0.0001**
Treatment *Sub-treatment	168.328	6	28.055	3.120	**0.021**
Error	215.794	24	8.991		
*Tests of Within-Subjects Effects*					
Time	146.841	1.998	73.489	19.221	**<0.0001**
Time* Treatment	101.418	5.994	16.919	4.425	**0.001**
Time*Sub-treatment	227.023	3.996	56.809	14.858	**<0.0001**
Time*Treatment*Sub-treatment	46.403	11.989	3.871	1.012	0.453
Error(time)	183.354	47.955	3.823		

* Fraction values of degrees of freedom were corrected for sphericity using the Greenhouse-Geisser correction.

The repeated measures ANOVA of the per capita effect magnitude ANOVA (Table 3) revealed that there were significant differences between the sub-treatments “Predators+Parasitoids” and “Parasitoids”. There were also significant differences between the treatment “density 40” and the other initial densities “120, 240 and 400”. The within-subjects effects analysis was significant only for the interaction of time with sub-treatment.

**Table 3:** Repeated-measures two-way ANOVA of the per capita effect magnitude with treatment (initial aphid densities), sub-treatment (“Predators”, “Predators+Parasitoids” and “Parasitoids”), and time of the counting as main effects.

Source		Type III Sum of Squares	df*	Mean Square	F	Sig.
*Tests of Between-Subjects Effects*						
Intercept	120.397		1	120.397	89.013	**<0.0001**
Treatment	86.133		3	28.711	21.227	**<0.0001**
Sub-treatment	15.000		2	7.500	5.545	**0.010**
Treatment*Sub-treatment	16.067		6	2.678	1.980	0.109
Error	32.462		24	1.353		
*Tests of Within-Subjects Effects*						
Time		0.996	1.295	0.769	1.207	0.295
Time*Treatment		2.252	3.886	0.579	0.909	0.468
Time*Sub-treatment		7.621	2.591	2.941	4.616	**0.011**
Time*Treatment*Sub-treatment		5.609	7.773	0.722	1.132	0.370
Error(time)		19.810	31.090	0.637		

* Fraction values of degrees of freedom were corrected for sphericity using the Greenhouse-Geisser correction.

The aphid numbers recorded at each of the three counts are shown in Fig. 2. At the first count (Fig. 2A, Table 3) differences were only marginally significant. In the sub-treatment “Predators” density 40 the effect on aphid numbers was less than in the other sub-treatments. There were no differences between the treatments densities 120 and 240. In the treatment density 400 there were significantly fewer aphids in those with predators than with parasitoids, but not those with both predators and parasitoids.

When the first larvae and parasitoid mummies started to appear (Fig. 2B, Table 4), there were no differences between the treatments 40, 120 and 240. In the treatment density 400 the number of aphids in those with predators was significantly different from those recorded in the other sub-treatments.

**Table 4:** One way ANOVA of 3 different counts (first count, time of first larvae and mummies appeared and last count).

		Sum of Squares	df	Mean Square	F	Sig.
*ANOVA One-way first counting*						
40	Between Groups	4.845	2	2.423	5.073	**0.051**
	Within Groups	2.865	6	0.478		
	Total	7.711	8			
120	Between Groups	3.432	2	1.716	4.124	0.075
	Within Groups	2.497	6	0.416		
	Total	5.929	8			
240	Between Groups	3.348	2	1.674	1.543	0.288
	Within Groups	6.511	6	1.085		
	Total	9.859	8			
400	Between Groups	22.086	2	11.043	5.248	**0.048**
	Within Groups	12.626	6	2.104		
	Total	34.713	8			
*ANOVA One-way first larvae and mummies*						
40	Between Groups	5.724	2	2.862	2.721	0.144
	Within Groups	6.312	6	1.052		
	Total	12.037	8			
120	Between Groups	0.562	2	0.281	0.336	0.727
	Within Groups	5.011	6	0.835		
	Total	5.573	8			
240	Between Groups	6.900	2	3.450	2.486	0.164
	Within Groups	8.327	6	1.388		
	Total	15.228	8			
400	Between Groups	58.411	2	29.205	27.648	**0.001**
	Within Groups	6.338	6	1.056		
	Total	64.749	8			
*ANOVA One-way last counting*						
40	Between Groups	47.911	2	23.956	2.192	0.193
	Within Groups	65.585	6	10.931		
	Total	113.497	8			
120	Between Groups	44.940	2	22.470	5.020	**0.052**
	Within Groups	26.859	6	4.476		
	Total	71.798	8			
240	Between Groups	86.371	2	43.186	171.598	**<0.0001**
	Within Groups	1.510	6	0.252		
	Total	87.881	8			
400	Between Groups	94.839	2	47.419	292.860	**<0.0001**
	Within Groups	0.972	6	0.162		
	Total	95.810	8			

At the last count (Fig. 2C, Table 4) with the exception of the lowest treatment the number of aphids in the “Predators” and “Predators+Parasitoids” sub-treatments were significantly lower than in the sub-treatments with “Parasitoids”.

**Figure 3:**
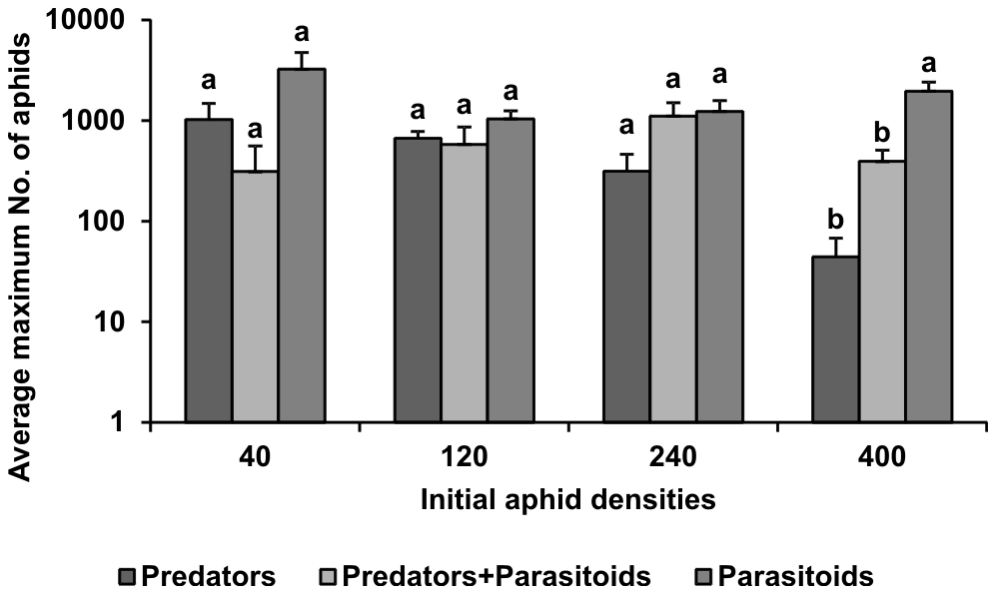
Average (±SE) maximum number of aphids recorded in each treatment. For each of the initial numbers of aphids used (40, 120, 240, 400 - indicated on the horizontal axis) different letters at the tops of the columns indicate significant differences between the means recorded in the different sub-treatments.

The results of the one-way ANOVA of the maximum number of aphids recorded in each sub-treatment (Fig. 3, Table 5) revealed that there were only significant differences at the highest density (400), “Predators” and “Predators+Parasitoids” sub-treatment had less aphids then “Parasitoids” but there were no differences between both.

**Table 5:** One-way ANOVA of the maximum aphid numbers recorded within the different treatments (initial aphid densities) and sub-treatments (“Predators”, “Predators+Parasitoids” and “Parasitoids”).

		Sum of Squares	df	Mean Square	F	Sig.
40	Between Groups	1.390E^+07^	2	6.951E^+06^	2.804	0.138
	Within Groups	1.488E^+07^	6	2.479E^+06^		
	Total	2.878E^+07^	8			
120	Between Groups	362546.889	2	181273.444	1.313	0.336
	Within Groups	828113.333	6	138018.889		
	Total	1.191E^+06^	8			
240	Between Groups	1.483E^+06^	2	741360.778	2.496	0.163
	Within Groups	1.782E^+06^	6	296992.889		
	Total	3.265E^+06^	8			
400	Between Groups	6.176E^+06^	2	3.088E^+06^	13.521	**0.006**
	Within Groups	1.370E^+06^	6	228379.333		
	Total	7.546E^+06^	8			

## Discussion

Here we demonstrate that the population dynamics of a system consisting of 2 natural enemies and a shared prey is strongly affected by the initial densities of the species in question and that these differences are not only quantitative but also qualitative. For example, during the first counting, aphids were least suppressed in the “Predators” sub-treatment at density 40, but most effectively suppressed in the same sub-treatment at density 400. Similarly, when the first larvae and mummies appeared, in the “Predators” sub-treatment aphids were suppressed significantly more than in other sub-treatments at density 400, but not so in all other densities, etc. Therefore, for example, the answer to a very practical question “is it better to use only predators, or a combination of predators and parasitoids to reduce the number of aphids below the economic threshold in the system studied?” strongly depends on whether low or high numbers of aphids and natural enemies are used. To give an example, Ferguson and Stiling [47] only used one set of starting densities of natural enemies and conclude that parasitoids alone are more effective than predators or a combination of both predators and parasitoids. Would this hold, if they used other initial densities? Our longer-term experiments indicate that it may not: in our case, ladybirds were more effective than parasitoids when there were high numbers of aphids than when there were initially few aphids. Thus, for determining the optimal strategy for biocontrol it is not sufficient to do experiments in which only the presence/absence of species is varied as the effect of varying absolute numbers of individuals must also be considered. The same is true, when considering the interactions between several species.

Considering not only relative but also absolute densities is important, since if negative interactions become more common with increase in enemy biodiversity, then it is not clear whether or not biocontrol strategies should include a greater species richness, especially because negative interactions among natural enemies can reduce their ability to suppress pest populations [29], [48]. It may also be important to distinguish natural from agricultural ecosystems as increase in diversity may not in all cases be the best option for the latter, as indicated by the results of this study. However, diversity can be advantageous if there is niche separation between the predators [49].

Our experiments revealed also some specific issues for the system used. The parasitoids we used were disturbed by the presence of high numbers of ladybirds and conspecifics, which increased the possibility of encounters among them resulting in intraguild-predation, competition or behavioural changes (e.g., parasitoids may be more reluctant to lay eggs in the presence of predators, even when aphid densities are high, which elicits a strong defensive behaviour in aphids against parasitoids). Mackauer and Völkl [50] report that aphidiid wasps reduce the incidence of attack by hyperparasitoids by usually laying their eggs in several host patches. Also, Taylor et al. [41] showed that cues from predators disturb parasitoid behaviour, in that aphid parasitoids encounter and oviposit in fewer aphids in the presence or recent presence of a predator. Thus, avoidance of potential predators may result in non-optimal foraging and reduced resource utilisation [51]. In our study the parasitoids were less able to suppress aphid populations, probably because of the reasons stated above. However, the negative effects of predator-parasitoid or parasitoid-parasitoid interactions might be less important in natural situations, than in cage experiments, as the growth rates and peak densities of aphid populations within cages are usually larger than those in uncaged populations [52]. Parasitoids are also more sensitive than predators to the defensive mechanisms of aphids, such as release of alarm pheromone [53], [54], body shaking, kicking off parasitoids, walking away [55] or clustering together [56], [57].

Our results also strongly indicate that the outcome strongly depends on the duration of the experiment. The time scale of most empirical studies is short, typically quantifying predation rates in one generation. In most studies the population growth of prey is only recorded over the period of time it takes the predator to complete one generation. In contrast, models focus on prey and predator population densities at equilibrium, typically after many predator and prey generations [18]. Thus, we should be cautious in drawing conclusions based on experiments that last only a few days and in using this type of data in general predictive models. Long-term experiments should be preferred in such cases. Also, more theoretical work could emphasize population dynamics away from equilibrium [58].
